# Diversity of arsenite oxidizing bacterial communities in arsenic-rich deltaic aquifers in West Bengal, India

**DOI:** 10.3389/fmicb.2014.00602

**Published:** 2014-11-21

**Authors:** Devanita Ghosh, Punyasloke Bhadury, Joyanto Routh

**Affiliations:** ^1^Integrative Taxonomy and Microbial Ecology Research Group, Department of Biological Sciences, Indian Institute of Science Education and Research-KolkataNadia, India; ^2^Department of Thematic Studies- Environmental Change, Linköping UniversityLinköping, Sweden

**Keywords:** Arsenic, aquifer, arsenite oxidation, *aioA* gene, phylogeny

## Abstract

High arsenic (As) concentration in groundwater has affected human health, particularly in South-East Asia putting millions of people at risk. Biogeochemical cycling of As carried out by different bacterial groups are suggested to control the As fluxes in aquifers. A functional diversity approach in link with As precipitation was adopted to study bacterial community structures and their variation within the As contaminated Bengal Delta Plain (BDP) aquifers of India. Groundwater samples collected from two shallow aquifers in Karimpur II (West Bengal, India), during years 2010 and 2011, were investigated to trace the effects immediately after monsoon period (precipitation) on community structure and diversity of bacterial assemblages with a focus on arsenite oxidizing bacterial phyla for two successive years. The study focused on amplification, clone library generation and sequencing of the arsenite oxidase large sub-unit gene *aioA* and 16S rRNA marker, with respect to changes in elemental concentrations. New set of primers were designed to amplify the *aioA* gene as a phylogenetic marker to study taxonomically diverse arsenite oxidizing bacterial groups in these aquifers. The overall narrow distribution of bacterial communities based on *aioA* and 16S rRNA sequences observed was due to poor nutrient status and anoxic conditions in these As contaminated aquifers. *Proteobacteria* was the dominant phylum detected, within which *Acidovorax, Hydrogenophaga, Albidiferax, Bosea*, and *Polymorphum* were the major arsenite oxidizing bacterial genera based on the number of clones sequenced. The structure of bacterial assemblages including those of arsenite oxidizing bacteria seems to have been affected by increase in major elemental concentrations (e.g., As, Fe, S, and Si) within two sampling sessions, which was supported by statistical analyses. One of the significant findings of this study is detection of novel lineages of 16S rRNA-like bacterial sequences indicating presence of indigenous bacterial communities BDP wells that can play important role in biogeochemical cycling of elements including As.

## Introduction

Arsenic (As) contamination in groundwater is a major problem in drinking water supplies in many countries (Nickson et al., 2000; Bhattacharya et al., 2001). The fertile deltaic plains drained by three major rivers Ganges, Brahmaputra and Meghna and covering an area of ca. 105,000 km^2^ in India and Bangladesh, are one of the worst As affected regions in the world (Mukherjee and Bhattacharya, 2001). This region is largely used for agriculture and there is a significant influence of monsoon on seasonal crops. Nearly 70 million people in this region are exposed to various As related chronic health problems from drinking As-rich groundwater, which poses major environmental challenge (Bhattacharya et al., 2002; Guha Mazumder, 2003).

Alluvial sediments and groundwater in the Bengal Delta Plain (BDP) aquifers are widely reported to have high levels of As (Bhattacharya et al., 1997; Nickson et al., 2000; Mukherjee and Bhattacharya, 2001; Bhattacharya et al., 2001; McArthur et al., 2004; Hossain et al., 2005). It is hypothesized that As is released in these aquifers because of: (1) oxidation of As-rich pyrites in sediments, (2) reductive dissolution of iron-hydroxides, and (3) exchange of P and As ions in fertilizers (Bhattacharya et al., 1997; Nickson et al., 2000; McArthur et al., 2004; Silver and Phung, 2005). Notably, the second hypothesis is widely accepted by most researchers; it indicates the vital role of microorganisms in biogeochemical cycling of As in the sub-surface. Microbes are involved in four major reactions that affect As cycling: oxidation, reduction, methylation and demethylation; the formation of organo-arsenicals however does not play a major role in As cycling (Oremland and Stolz, 2003). The prokaryotes involved in As oxidation include bacteria and archaea. Arsenite{As(III)} oxidizing bacteria are broadly classified into two categories: Chemolithoautotrophic Arsenite Oxidizers (CAOs) and Heterotrophic Arsenite Oxidizers (HAOs) (Oremland and Stolz, 2005). Bacterial oxidation of As(III) involves a key enzyme As(III) oxidase, which contains a Rieske sub-unit (smaller sub-unit *aioB*) and a molybdopterin binding sub-unit (larger sub-unit *aioA*; Lebrun et al., 2006; Lett et al., 2012). In addition, the arsenite oxidase enzyme possesses three more sub-units: *aioS* (sensor histidine kinase), *aioR* (transcriptional regulator) and *aioX* (oxy-anion binding protein; Lett et al., 2012). The expression of As(III) oxidase enzyme is stringently regulated by As concentration (Katsoyiannis and Zouboulis, 2006). The molybdopterin binding large sub-unit *aioA* in As(III) oxidase has an iron-sulfur [3Fe-4S] binding conserved motif, and another consensus motif enabling it to be used to study molecular phylogeny and distribution of As(III) oxidizing bacteria throughout the world (Costello and Lidstrom, 1999). Thus, polyphyly of As(III) oxidizing bacteria can be closely investigated using potential molecular markers such as *aioA*, within or between the different sites (Quemeneur et al., 2008; Salmeron et al., 2011).

Sedimentary organic matter transported into groundwater through rainfall (recharge) has been suggested to play a crucial role in sustaining the microbial communities involved in biogeochemical cycling of various elements including As (McArthur et al., 2004; Rowland et al., 2006). Previous investigations undertaken in the Bengal deltaic sediments had indicated the presence of petroleum derived hydrocarbons playing a crucial role in microbial mediated As dissolution (Rowland et al., 2006; Héry et al., 2010). Previously, researchers had mostly focused on unraveling the community diversity of As(III) oxidizing bacteria based on *aioA* gene fragments in contaminated mining sites, microbial mats and geothermal springs (e.g., Quemeneur et al., 2008; Salmeron et al., 2011). There is a notable omission in the study of diversity of As(III) oxidizing bacteria in As-rich potable water resources. Moreover, it must be mentioned that molecular tools involved in detection of As(III) oxidizing bacteria is in its early stages of development and therefore, the number of available *aioA* gene sequences in published databases are very limited from the context of As contaminated aquifers (in particular from BDP aquifers).

Recently, phylogeny of bacterial communities using 16S rRNA signature in As contaminated BDP aquifers in Bangladesh were investigated (Sultana et al., 2011). However, the authors did not specifically report As(III) oxidizing bacterial communities as a part of their study. Likewise, very little is known about bacterial community structure and diversity in the BDP aquifers in West Bengal, India-a known As “hot-spot” (Bhattacharya et al., 2002; Mukherjee and Fryar, 2008; Biswas et al., 2012). Hence, the key questions in this study are: (1) what are the major arsenite oxidizing bacterial groups present in BDP aquifers controlling distribution of As based on amplification, sequencing and phylogeny of *aio*A gene, (2) how does diversity and richness of bacterial assemblages change, specifically arsenite oxidizing bacterial assemblages, following changes in groundwater elemental concentrations immediately after monsoon for two successive years in BDP aquifers in West Bengal (India) based on the functional gene (*aio*A) and 16S rRNA sequencing approach. To the best of our knowledge, this is the first study of its kind where phylogenetic assemblages of As(III) oxidizing bacteria, and the effect immediately after monsoon on bacterial community structures in aquifers for the years studied, with special emphasis on arsenite oxidizing bacterial groups have been attempted. In West Bengal, where the As problem is critical and suitable remediation schemes are largely unsuccessful (Hossain et al., 2005), detailed investigations on bacterial communities specifically the As(III) oxidizing bacteria may provide better understanding of ongoing biogeochemical processes associated with As cycling in order to implement suitable and effective remediation methods.

## Study area

**Figure 1:**
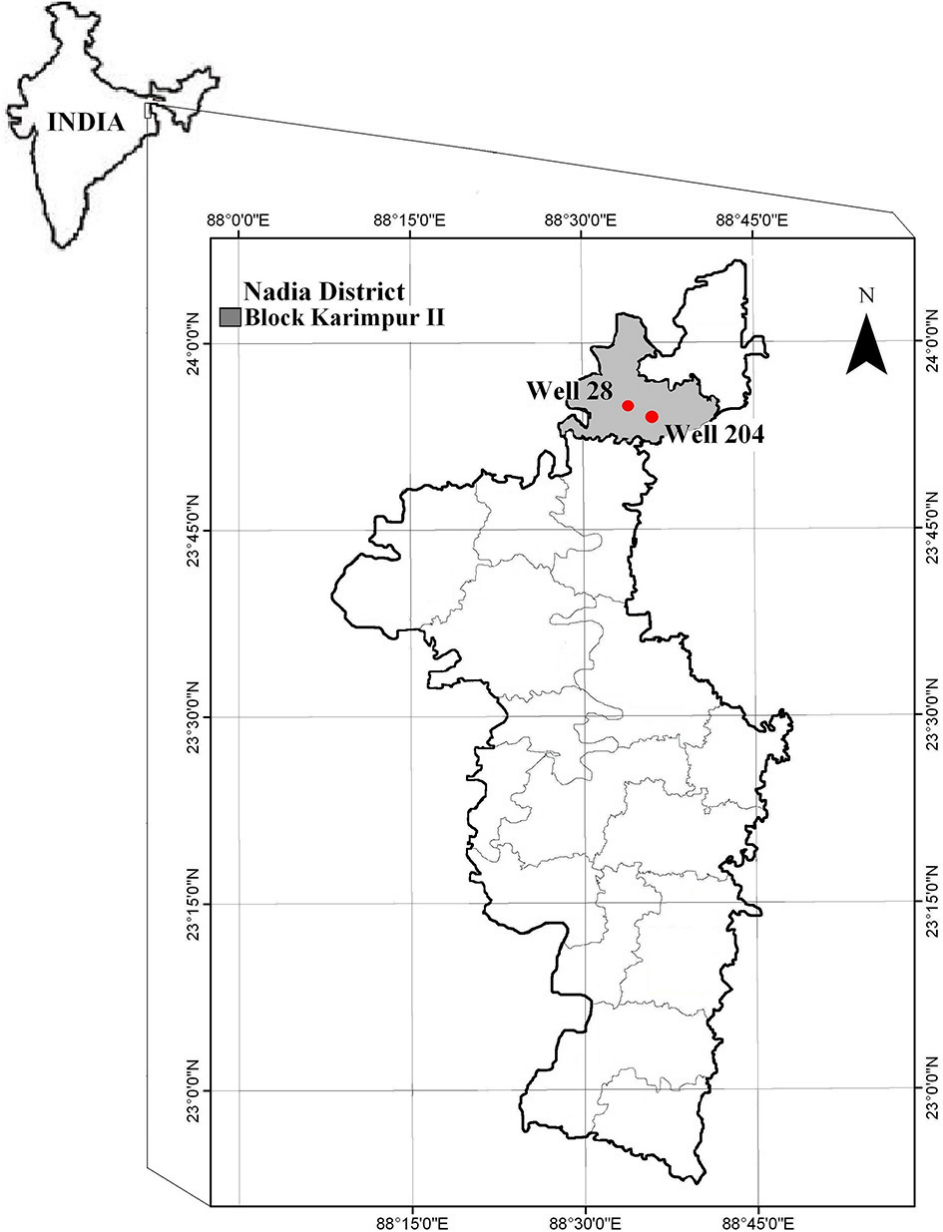
**Map showing sampling locations in Nadia district (West Bengal, India)**.

Various districts in West Bengal (India) are tagged as As “hot-spots” (Chowdhury et al., 2000; Bhattacharya et al., 2001, 2002; Nath et al., 2005), because of high As concentration in groundwater. The sampling site, Karimpur II block in Nadia district in West Bengal, is one of the worst As affected areas in West Bengal (Figure 1). A survey conducted during the year 2002–06 by PHED (Public Health Engineering Department, West Bengal) and UNICEF (Summary of water quality status^1^) indicated that 46% of the pre-installed tube wells in this block have As levels higher than 50 μg/l (Supporting information provided in Table S1). Sedimentary sequences in these alluvial aquifers comprise of intercalating layers of clay, silt and gray sand fining downward. This type of sub-surface lithology widely recognized in this region are connected to gray sand aquifers (GSA; Rowland et al., 2006). The average temperature in the district is 42°C during summer and 9°C in winter (Statistical Handbook, 2010), and the average rainfall is 99.66 mm. In 2010, the average rainfall was higher (117.7 mm) and the water table was between 2 and 6 m. However in 2011 average rainfall was significantly less than previous year (70.5 mm) (District rainfall five year reports, 2008–12^2^).

## Methods

### Sample collection and environmental DNA extraction

We conducted a reconnaissance survey in 2010 targeting some of the As-rich boreholes that had been previously identified by PHED, for undertaking extraction of environmental DNA samples. During the pre-screening exercise, only two out of eight gray sand shallow aquifer boreholes 28 (N 23°55.064′, E 088°33.350′) and 204 (N 23°56.352′, E 088°33.814′) indicated positive results for the presence of *aioA* gene, and these boreholes, were subsequently pursued to determine microbial community structures and major elemental concentrations. Sampling was undertaken in September 2010 immediately after the end of monsoon and in December 2011 after the end of monsoon (the monsoons started late and ended early in 2011). Groundwater was collected from these two wells after pumping out roughly three times the well volume of groundwater to ensure the collection of fresh groundwater sample. Environmental parameters namely pH (Eco testr pH 2), temperature, conductivity and TDS (Sartorius, PY-Y12) were measured immediately after collecting the sample. Two sets of water samples (50 ml) were collected from both wells in sterile falcon tubes, and used to determine the concentration of dissolved elements. One set was immediately acidified by adding 2–3 drops of concentrated HNO_3_ (Merck) and then filtered (0.45 μm). Dissolved elements in the water samples were analyzed on an ICP-MS (Perkin Elmer NexION 300D). The detection limit of the elemental analysis was at μg/l level.

For microbial assays absolute molecular grade ethanol (Merck) was added to groundwater samples (2 liters) to a final concentration of 2% immediately after collection in order to limit microbial activity and prevent denaturation of nucleic acids, which may lead to substantial changes in microbial community structure during transportation to laboratory (Stein et al., 2013). The samples were immediately transported to laboratory and were passed through 0.22 μm Sterivex filters (Millipore) using a peristaltic pump for concentrating the biomass. The filters were stored overnight at −20°C temperature and environmental DNA was extracted the very next day using standard published protocols (Tringe and Rubin, 2005; Liles et al., 2009). After extraction, the DNA was quantified on an UV-Vis spectrophotometer (Beckman DU730).

### Designing primers for *aioA* gene fragment amplification

To design a new set of *aioA* primers, 67 *aioA* full length amino acid sequences (both putative and verified) were downloaded from GenBank/EMBL/PDB databases and aligned in ClustalW (Larkin et al., 2007) for identification of conserved domains. A [3Fe-4S] cluster binding region was found to be conserved across the set of aligned sequences. Hence, this region was selected for designing a new forward primer *aioA*F 5′CCACTTCTGCATCGTGGG 3′ and a consensus motif IIWGNDN was targeted based on the newly designed reverse primer *aioA*R 5′ TGTCGTTGCCCCAGATGA 3′. The primers were subsequently checked in Primer-BLAST version 2.2.3 (Rozen and Skaletsky, 2000) for self-complementation, low GC content and low T_m_ value to ensure sufficient thermal window for efficient annealing. The size of *aioA* amplicons generated using this set of primer was approximately 1114 bp. The specificity of the newly designed primers were tested on genomic DNA extracted from 4 previously reported arsenite oxidizing bacterial genera *Thiomonas arsenivorans* strain b6 (Battaglia-Brunet et al., 2006a), *Leptothrix* sp. strain S1.1 (Battaglia-Brunet et al., 2006b), *Variovorax* sp. strain 4.2 (Battaglia-Brunet et al., 2006b), *Herminiimonas arsenicoxydans* strain ULPAs1 (Weeger et al., 1999), and 4 bacterial strains isolated in our laboratory from the arsenic contaminated water in BDP wells 28 and 204. These were *Acinetobacter lwoffii* strain BDP2, *Hydrogenophaga atypica* strain BDP10, *Hydrogenophaga bisanensis* strain BDP20 and *Acidovorax facilis* strain BDP24 (Ghosh et al., unpublished). Incidentally, the whole genome of *H*. *arsenicoxydans* strain ULPAs1 has been also sequenced (Muller et al., 2007). Additionally, the *aio*A primers were also tested on the genomic DNA extracted from non-arsenic oxidizing bacteria such as *Escherichia coli* and *Bacillus subtilis*.

### PCR amplification of partial *aioA* gene and 16S rRNA fragments

Amplification of the partial *aioA* gene fragments was undertaken from environmental DNA extracted from 2010 to 2011 groundwater samples using the newly designed *aioA* primers. Each PCR reaction consisted of 0.5 μL (~20 ng) DNA template, 5.0 μL 10X Dream *Taq* buffer, 5.0 μL dNTPs (final concentration 0.2 mM), 5.0 μL MgCl_2_ (final concentration 2.0 mM), 0.5 μL each of the newly designed *aioA* primers (final concentration 5 μM), 0.5 μL BSA (1 mg/ml), 0.5 μL DNA Dream *Taq* polymerase (5 U/μL) (Fermentas) and nuclease free water to make a final volume of 50 μL. PCR conditions were as follows: initial denaturation at 95°C for 10 min, 35 cycles of 95°C for 1 min, 70°C for 1 min, 72°C for 2.30 min, and final extension at 72°C for 10 min. The annealing temperature optimum for *aio*A amplicon yield that was initially selected was based on the gradient PCR approach. For amplification of *aio*A genes from eight arsenite oxidizing bacterial isolates as well as from non-arsenite oxidizing bacteria (as mentioned earlier), the same PCR conditions were followed.

PCR amplification of the bacterial 16S rRNA fragments was undertaken from environmental DNA using standard eubacterial primers namely: Fc27 (5′-AGAGTTTGATCCTGGCTCAG-3′) and RC1492 (5′-TACGGCTACCTTGTTACGACTT-3′) (Lane, 1991). Each PCR reaction consisted of 0.5 μL DNA Dream *Taq* polymerase (5 U/μL) (Fermentas), 5.0 μL 10X Dream *Taq* buffer, 5.0 μL dNTPs (final concentration 0.2 mM), 5.0 μL MgCl_2_ (final concentration 2.0 mM), 0.5 μL of each primers (final concentration 5 μM), 0.5 μL (~20 ng) DNA template, 0.5 μL BSA (1 mg/ml), and nuclease free water to make a final volume of 50 μL. The PCR conditions applied were as follows: initial denaturation at 95°C for 10 min, 36 cycles of 95°C for 1 min, 55°C for 1 min, 72°C for 3.30 min, and final extension at 72°C for 10 min.

All the PCR reactions (*aioA* and 16S rRNA fragments) were performed in triplicates and subsequently, pooled together and purified using the Gel Purification Kit (Qiagen) as per manufacturer's instructions.

### Clone library and DNA sequencing

Purified PCR products were cloned using pGEM^®^-T Easy vector system (Promega) following the manufacturer's instructions. Plasmid DNA containing inserts were sequenced in both directions using SP6 and T7 primers in an ABI Prism 3730 Genetic Analyzer based on BigDye Terminator chemistry. The four libraries for *aioA* gene fragment have been referred to as 28WR2010, 28WR2011, 204WR2010, and 204WR2011 throughout the paper. Similarly, the 16S rRNA libraries have been referred to as 28WS2010, 28WS2011, 204WS2010, and 204WS2011.

### Phylogenetic analysis of generated sequences

Sequence chromatograms were checked manually for miss-spaced peaks, double peaks, and peak shifts using BioEdit version 7.1.3 (Hall, 1999). The *aioA* nucleotide sequences were translated into amino acid sequences in EMBOSS Transeq (Rice et al., 2000), and subsequently compared against protein databases (GenBank/EMBL/PDB) using the blastp tool (Camacho et al., 2008). The 16S rRNA nucleotide sequences were additionally checked for chimera using Bellerophon (Huber et al., 2004), and chimeric sequences were excluded from downstream analyses. The16S rRNA sequences were subsequently compared against nucleotide databases (GenBank/EMBL/DDBJ) using the blastn tool (Camacho et al., 2008). The top 10 published cultured and uncultured bacterial *aioA* amino acid sequences that overlapped with the *aioA* sequences generated in this study based on the blastp results were aligned using ClustalW (Larkin et al., 2007). The *aioA* alignment consisted of 229 sequences generated from this study, and 17 published *aioA* sequences of uncultured and cultured bacteria from the databases. For 16S rRNA sequences, top 10 published cultured and uncultured bacterial 16S rRNA nucleotide sequences which overlapped with 16S rRNA sequences generated from this study were also aligned using ClustalW. The alignment consisted of 169 16S rRNA sequences generated from this study and 59 published uncultured and cultured bacterial 16S rRNA sequences from the databases. Both the alignments were manually checked for errors in Seaview (version 4.0; Gouy et al., 2010). A neighbor joining (NJ) method (Saitou and Nei, 1987) based on the JTT model (Jones et al., 1992) was used to construct the phylogenetic tree for *aioA* in MEGA version 5.0 (Tamura et al., 2011). In case of 16S rRNA phylogeny, NJ method based on Kimura 2 parameter model (Kimura, 1980) was used for constructing the tree. Bootstrap test was conducted for both the trees to get the best topology from 50% majority rule consensus tree (Felsenstein, 1985). To root the *aioA* and 16S rRNA phylogenetic trees, the amino acid sequence of As(III) oxidase large sub-unit of *Thermus aquaticus*Y51MC23 (Acc. No. EED09253) and the 16S rRNA gene sequence of *Methanobrevibacter smithii* (Acc. No. U55235) were taken as outgroups, respectively. The sequences of *aioA* clones and 16S rRNA clones generated in this study have been submitted to GenBank and their accession numbers are from KF840950 - KF841347. All the eight *aioA* sequences obtained from cultured arsenic oxidizing bacterial isolates have been submitted to GenBank (accession numbers KM884948 - KM884955).

### Statistical analyses

For taxonomic analysis and comparison of temporal trends among *aioA* and 16S rRNA clone libraries, the generated sequences were grouped into operational taxonomic units (OTUs) based on 2% cut-off at amino acid level for *aioA* sequences and 3% cut-off at nucleotide level for 16S rRNA sequences using DOTUR (Schloss and Handelsman, 2005). Shannon-Wiener indices (H′) were also calculated in DOTUR for measurement of biodiversity of the pooled *aioA* and 16S rRNA clone libraries as well as for individual clone library. Total expected numbers of OTUs in each clone library was calculated using non-parametric richness estimator Chao 1 in DOTUR. Phylotype (or OTUs) frequency curves were prepared to determine the frequency of OTUs across the clone libraries. Rarefaction analysis was undertaken in DOTUR to compare the diversity of clone libraries.

Principal Component Analysis (PCA) is a widely used for analyzing variance among environmental parameters in microbial ecology studies (Dollhopf et al., 2001). In this study, to understand the role of various physicochemical variables like pH, groundwater temperature, ionic conductivity, total dissolved solids (TDS) and concentration of various major elements on the distribution of *aioA* gene and 16S rRNA OTUs, a PCA was carried out in PRIMER v 6.0 (Clarke and Gorley, 2006). To minimize the variation in different data set, square root transformation was done prior to PCA. The OTUs were superimposed as bubbles to determine the role of these variables in shaping the OTU trends (Figure S3 in supporting information).

## Results

### Trace metal analyses

The pH and ionic conductivity in groundwater samples from both wells increased in 2011 in comparison with 2010 (Table 1). Arsenic concentration exceeded the WHO limit of (10 μg/l; WHO, 2011) for safe drinking water; we found the As concentration to be >30 μg/l in all samples from both sampling events. Arsenic concentration in well 28 was 35 μg/l in 2010, which increased to 55 μg/l in 2011. Similarly in well 204, As concentration was 37 μg/l in 2010, which subsequently increased to 117 μg/l in 2011. Iron concentration also increased between the year 2010 and 2011. In 2010, Fe concentration in well 28 was 1 mg/l, which increased to 1.65 mg/l in 2011. In well 204, Fe concentration was 0.39 mg/l, which increased to 3.45 mg/l in 2011. In both cases, Fe exceeded the WHO guideline limit of 0.009 mg/l in drinking water (WHO, 2011). We also observed an increase in silica (Si) concentration in groundwater samples collected in 2011 compared to 2010. However, such temporal changes were not significant for other elements like magnesium (Mg), manganese (Mn), molybdenum (Mo) and potassium (K) in both wells. The concentrations of various elements in groundwater samples are summarized in Table 2.

**Table 1:** **Physicochemical parameters of collected water samples from two wells 28 and 204 for the two consecutive years (2010 and 2011) from Karimpur II, West Bengal, India**.

**Well Number**	**pH**	**Ionic Conductivity (μSiemens)**	**TDS (mg/l)**	**Temperature (°C)**
Well No. 28 Year 2010^*^	6.9	673	336	24.7
Well No. 28 Year 2011^*^	7.3	717	358	22.3
Well No. 204 Year 2010^*^	6.8	741	373	24.4
Well No. 204 Year 2011^*^	7.3	758	379	23.4

* Sampling year.

**Table 2:** **Concentration of various interacting metals in collected water samples from two wells 28 and 204 for the two years (2010 and 2011)**.

**Well Number**	**Concentration of various metals**									
	As^*^	**Fe^‡^**	**Mg^‡^**	Mn^*^	Mo^*^	P^*^	S^*^	**Si^‡^**	**K^‡^**	**Na^‡^**
Well No. 28 Year 2010	35.0	1.00	24.7	690	2.00	420	20.0	1.13	3.56	2.60
Well No. 28 Year 2011	**55.0**	**1.65**	14.8	300	**3.00**	60.0	**470**	**6.35**	1.51	**8.70**
Well No. 204 Year 2010	37.0	0.39	25.7	370	3.00	230	20.0	1.02	2.70	2.14
Well No. 204 Year 2011	**117**	**3.45**	19.6	**480**	2.00	120	**410**	**6.23**	1.44	1.05

* Concentration in μg/l;

‡ concentration in mg/l Numbers in bold indicate inter-annual increase in concentration.

### Validation of new *aioA* primers

The newly designed *aioA* primers were tested for specificity by targeting genomic DNA extracted from published arsenite oxidizing bacterial strains such as *T. arsenivorans* strain b6 (Acc. No. KM884948), *Leptothrix* sp. strain S1.1 (Acc. No. KM884954), *Variovorax* sp. strain 4.2 (Acc. No. KM884949) and *H. arsenicoxydans* strain ULPAs1 (Acc. No. KM884955) and 4 bacterial strains isolated from BDP aquifers namely, *A. lwoffii* strain BDP2 (Acc. No. KM884950), *H. atypica* strain BDP10 (Acc. No. KM884951), *H. bisanensis* strain BDP20 (Acc. No. KM884952) and *A. facilis* strain BDP24 (Acc. No. KM884954). All the arsenite oxidizing bacterial isolates gave a positive amplification of ca. 1114 bp, and sequencing of the amplicons followed by pblast validation showed significant identity (99% and above) with published arsenite oxidizing bacterial *aio*A amino acid sequences available in GenBank/EMBL/PDB. For example, the *aio*A amplicons of *Variovorax* sp 4.2, *Leptothrix* sp. S1–1 and *H*. *arsenicoxydans* amplified using the new primer set showed 100% identity at the amino acid level with published *aioA* sequences of these three strains available in GenBank/EMBL indicating that the primers were gene specific. No amplification products were obtained when the primer set was tested on genomic DNA extracted from non-arsenite oxidizing bacteria such as *E*. *coli* and *B*. *subtilis*.

### Phylogenetic diversity analyses

**Figure 2:**
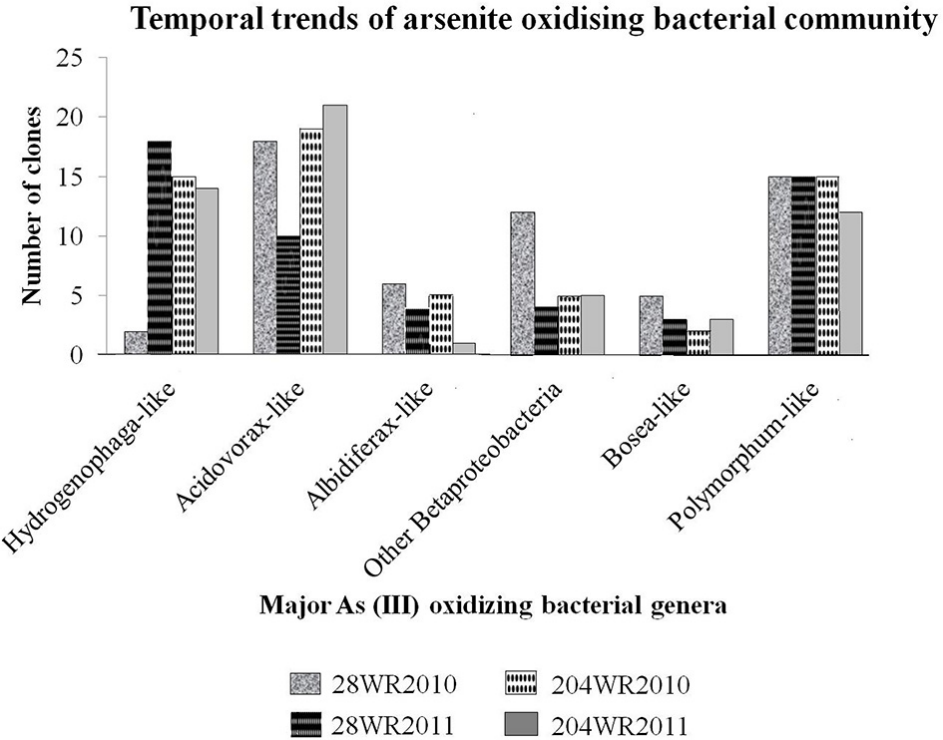
**Temporal trends of important As(III) oxidizing bacterial groups studied in the two wells 28 and 204 for two consecutive years (2010 and 2011)**.

To understand the effect of increasing concentration of different elements on bacterial communities, with a special emphasis on arsenite oxidizing bacterial groups based on *aio*A gene signature, a comparative approach was taken while taking into consideration varying precipitation between the sampled years immediately after monsoon. We monitored the change of *in situ* bacterial communities with focus on As (III) oxidizing bacterial groups from both wells (28 and 204) during years 2010 and 2011 in link with precipitation immediately after monsoon. A total of 229 *aioA* clones and 169 16S rRNA clones were sequenced from both wells. The temporal trends of As (III) oxidizing bacterial groups in these two wells, had been detailed in Figure 2.

The *aioA* clone libraries consisted of only As(III) oxidizing *Proteobacteria*-like sequences. The *aioA* clone libraries 28WR2010 (58 clones), 204WR2010 library (61 clones) and 204WR2011 (56 clones) showed 90–99% identity, whereas *aioA* clone library 28WR2011 (54 clones) showed 83–99% identity at the amino acid level with published *aioA* amino acid sequences represented by various cultured and uncultured *Alphaproteobacteria* and *Betaproteobacteria* from published databases (GenBank//EMBL/PDB). The highest and lowest identity scores for all *aioA* libraries at amino acid level have been summarized in Table S2 in supporting information.

The 16S rRNA clone libraries consisted dominantly of *Proteobacteria*-like sequences and few *Nitrospirae*-like sequences. Among the four 16S rRNA clone libraries, 28WS2010 (46 clones) showed 94–100% identity, 28WS2011 (38 clones) showed 95–100% identity, 204WS2010 (43 clones) showed 94–99% identity and 204WS2011 (42 clones) showed 94–100% identity. The similarity at nucleotide level with published cultured and uncultured bacterial 16S rRNA sequences belonged to *Alphaproteobacteria, Betaproteobacteria*, and *Gammaproteobacteria*. In the library 28WS2011, 4 clones (BDP28WS65, BDP28WS67, BDP28WS79, and BDP28WS80) showed 100% identity with the bacterium *Leptospirillum* sp. E4-L9 (Acc. No. HM769767) belonging to the phylum *Nitrospirae.* The highest and lowest identity scores for all the 16S rRNA clone libraries (at nucleotide level) have been detailed in Table S3 in supporting information.

Two phylogenetic trees were constructed based on the NJ method to understand the taxonomic affiliation of *aioA* sequences and 16S rRNA sequences with published cultured and uncultured bacterial *aioA* amino acid sequences and 16S rRNA sequences available in GeneBank/EMBL/DDBJ/PDB databases (Figures S1, S2a,b, respectively in supporting information).

The *aioA* phylogenetic tree formed two major clades represented by *Alphaproteobacteria* and *Betaproteobacteria* with strong bootstrap support (Figure S1 in supporting information). Only the major sub-clades have been discussed below. The largest clade *Betaproteobacteria* consisted of three sub-clades belonging to order *Burkholderiales*. The first and biggest sub-clade consisted of 75 *aioA* clones {28WR2010 (14 clones), 28WR2011 (22 clones), 204WR2010 (20 clones) and 204WR2011 (19 clones)} along with published *aioA* sequences represented by autotrophic As(III) oxidizing bacterium *Hydrogenophaga defluvii* (Acc. No. BAK39656) as well as uncultured bacterial *aioA* sequences targeted from As contaminated soils in Belgium and aquatic sediments in Japan. The second biggest sub-clade consist of 68 *aioA* clones {28WR2010 (18 clones), 28WR2011 (10 clones), 204WR2010 (19 clones) and 204WR2011 (21 clones)} along with published *aioA* sequences of the bacteria *Acidovorax* sp. NO-1 (Acc. No. ZP_09329325) and *Acidovorax* sp. 75 (Acc. No. ABY19324). The third sub-clade was represented by 16 *aioA* sequences {28WR2010 (6 clones), 28WR2011 (4 clones), 204WR2010 (5 clones) and 204WR2011 (1clone)} along with *aioA* sequence of iron-reducing bacterium *Albidiferax ferrireducens* (Acc. No. WP011465357) and several uncultured As(III) oxidizing bacterial sequences targeted previously from microbial mats in a Polish gold mine.

Under the *Alphaproteobacteria*-like clade, two sub-clades were observed in the *aioA* phylogenetic tree. The bigger sub-clade consisted of 57 clones {28WR2010 (15 clones), 28WR2011 (15 clones), 204WR2010 (15 clones) and 204WR2011 (12 clones)} along with published *aioA* amino acid sequences of petroleum hydrocarbon degrading bacterium *Polymorphum gilvum* SL003B-26A1 (Acc. No. YP004304060). The second sub-clade represented by 13 *aioA* clones {28WR2010 (5 clones), 28WR2011 (3 clones), 204WR2010 (2clones) and 204WR2011 (3 clones)} along with published *aioA* sequence of the autotrophic As(III) oxidizing bacterium *Bosea* sp. WAO (Acc. No. ABJ55855). All these sub-clades were supported by strong bootstrap values.

**Figure 3:**
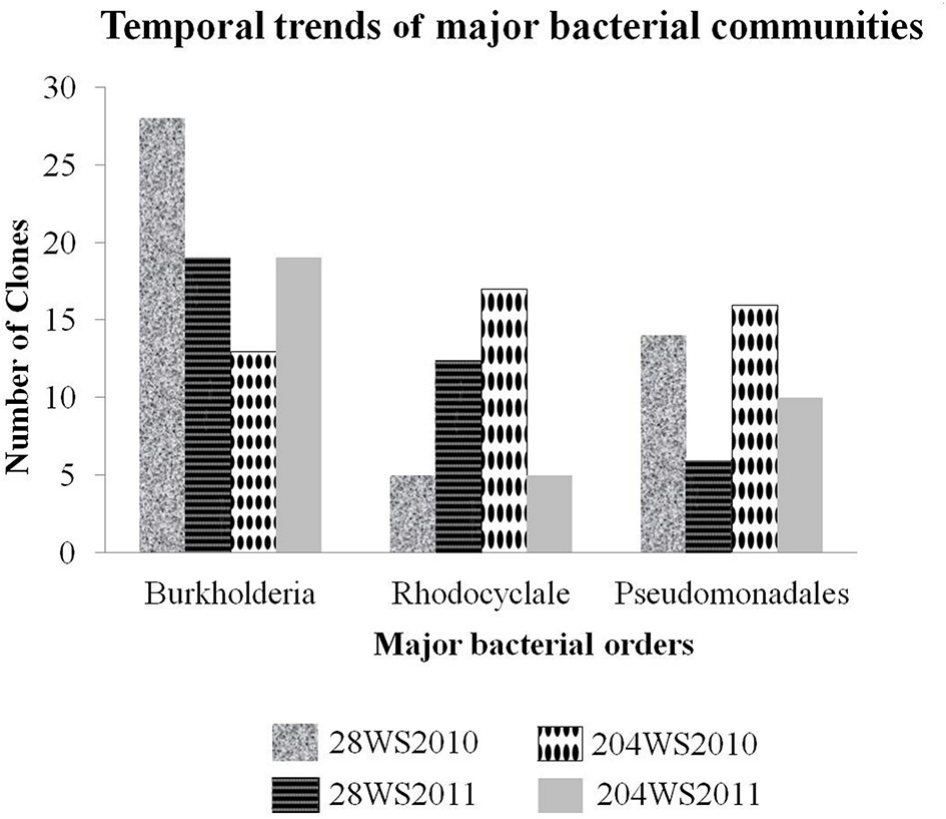
**Temporal trends of major bacterial groups studied in the two wells 28 and 204 for two consecutive years (2010 and 2011)**.

In the 16S rRNA phylogenetic tree, sequences generated from this study belonged to two major bacterial phyla namely, *Proteobacteria* and *Nitrospirae.* The temporal trends of major bacterial groups in these two wells, had been detailed in Figure 3. The phylum *Proteobacteria* consisted of sequences representing three classes *Alphaproteobacteria, Betaproteobacteria* and *Gammaproteobacteria* with strong bootstrap support (Figure S2a,b) in supporting information). The *Betaproteobacteria* consisted of two major clades represented by two orders namely *Burkholderiales* and *Rhodocyclales*. The first and the major clade (Figure S2a in supporting information) *Burkholderiales* consisted of 79 16S rRNA clones and were separated into three main sub-clades. The biggest sub-clade was represented by 73 16S rRNA clones {28WS2010 (28 clones), 28WS2011 (19 clones), 204WS2010 (7 clones) and 204WS2011 (19 clones)} along with the published 16S rRNA sequence of As(III) oxidizing bacteria such as: *Hydrogenophaga* sp. p3 (2011) (Acc. No. HQ652595), *Hydrogenophaga atypica* strain BSB 41.8 (Acc. No. NR029023) and *Hydrogenophaga* sp. AH-24 (Acc. No. AB300163) isolated from magnetite mine drainage, *Acidovorax* sp. BSB421 (Acc. No. Y18617) and several uncultured *Burkholderiales* bacterial clones including uncultured *Acidovorax* sp. clone OTU-29-AW (Acc. No. JQ624270) and uncultured *Variovorax* sp. clone 5.17m34 (Acc. No. JN679199) targeted previously in fresh water environments. Two sub-clades represented by 6 16S rRNA sequences from 204WR2010 clone library did not show any phylogenetic affiliation with published cultured and uncultured 16S rRNA sequences.

The second clade (Figure S2a in supporting information) *Rhodocyclale* consisted of two major sub-clades. The first one is represented by 8 16S rRNA clones {28WS2010 (2 clones), 28WS2011 (5 clones) and 204WS2010 (1clone)} along with published 16S rRNA sequence of bacterium, *Dechloromonas* sp. clone ECC3-2 (Acc. No. GU056291). The second sub-clade consisted of 31 16S rRNA clones {28WS2010 (3 clones), 28WS2011 (7clones), 204WS2010 (15 clones) and 204WS2011 (6 clones)} along with 16S rRNA sequences of cultured bacteria represented by *Zoogloea resiniphila* PIV-3C2w (Acc. No. AJ505853), *Zoogloea* sp. A5 (Acc. No. DQ342276), *Zoogloea resiniphila* strain DhA-35 (Acc. No. NR027188) and *Zoogloea oryzae* NBRC 102407 (Acc. No.AB681763) as well as with an iron-reducing enrichment culture clone HN104 (Acc. No. FJ269070) and various uncultured bacterial sequences targeted earlier from crude oil reservoirs and bacterioplanktonic assemblages.

The *Gammaproteobacteria*-like clade consisted of two major sub-clades represented by a single order *Pseudomonadales* (Figure S2b in supporting information). The first sub-clade consisted of 27 16S rRNA clones {28WS2010 (8 clones), 28WS2011 (6 clones), 204WS2010 (9 clones) and 204WS2011 (4 clones)} along with published 16S rRNA sequence represented by the bacterium *Acinetobacter* sp. TPR15 (Acc. No. EU373427), various uncultured *Acinetobacter* sp. 16S rRNA clones and other uncultured bacterial clones targeted previously in fresh water environments. The second sub-clade consisted of 19 16S rRNA clones {28WS2010 (5 clones), 28WS2011 (1clone), 204WS2010 (4 clones) and 204WS2011 (9 clones)} along with published 16S rRNA sequence from bacteria *Pseudomonas* sp. X5 (Acc. No. EU072020), *Pseudomonas* sp. Z64-1zhy (Acc. No. AM411070) and various uncultured bacterial 16S rRNA sequences targeted previously in fresh water environments, agricultural fields and petroleum reservoirs.

The *Alphaproteobacteria*-like clade is represented by only one 16S rRNA clone sequence (BDP204WS38) from 204WS2011 library along with published 16S rRNA sequence from the bacteria *Rhizobium selenitireducens* strain B1 (Acc. No. NR044216), *Agrobacterium* sp. enrichment cultures clone Van101 (Acc. No. HQ222282) and uncultured bacterial clone E13 (Acc. No. EU864455) earlier reported in industrial sources (Figure S2b in supporting information).

In the *Nitrospirae* like clade, only 4 16S rRNA clones from 204WS2011 library clustered with 16S rRNA sequence of *Leptospirillum* sp. E4-L9 (Acc. No. HM769767) isolated previously from a packed bed bioreactor used for ferrous iron bio-oxidation, with 100% bootstrap support (Figure S2b in supporting information).

### Rarefaction analysis and OTU calculations

**Figure 4:**
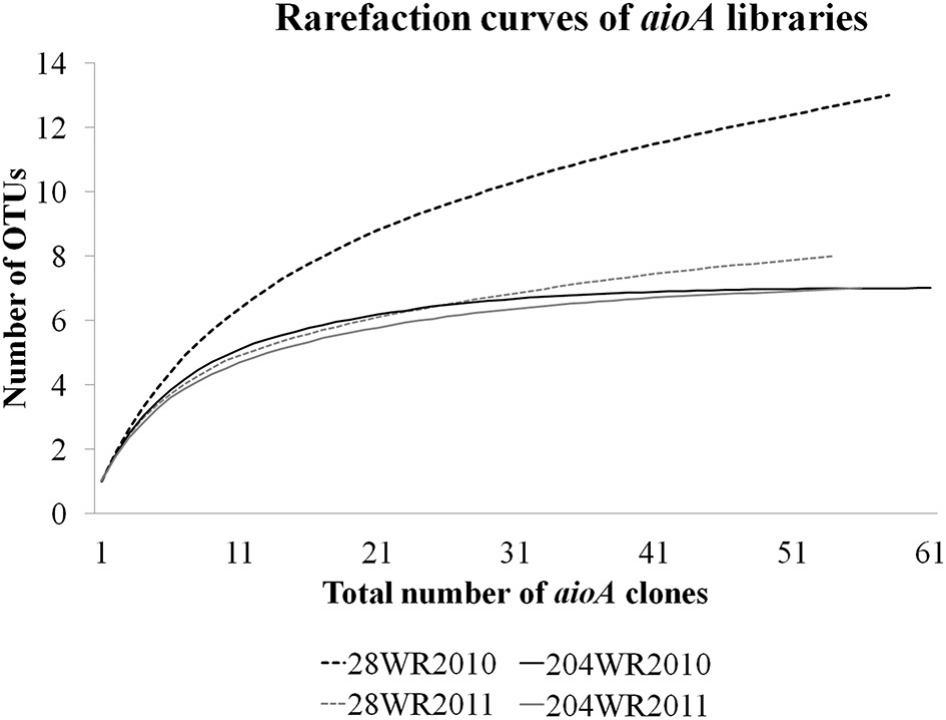
**Rarefaction curves from DOTUR analysis using furthest neighbor assignment algorithm with the *aioA* related amino acid sequences retrevied from wells 28 and 204 for the two years (2010 and 2011) at 0.02 evolutionary distance**.

**Figure 5:**
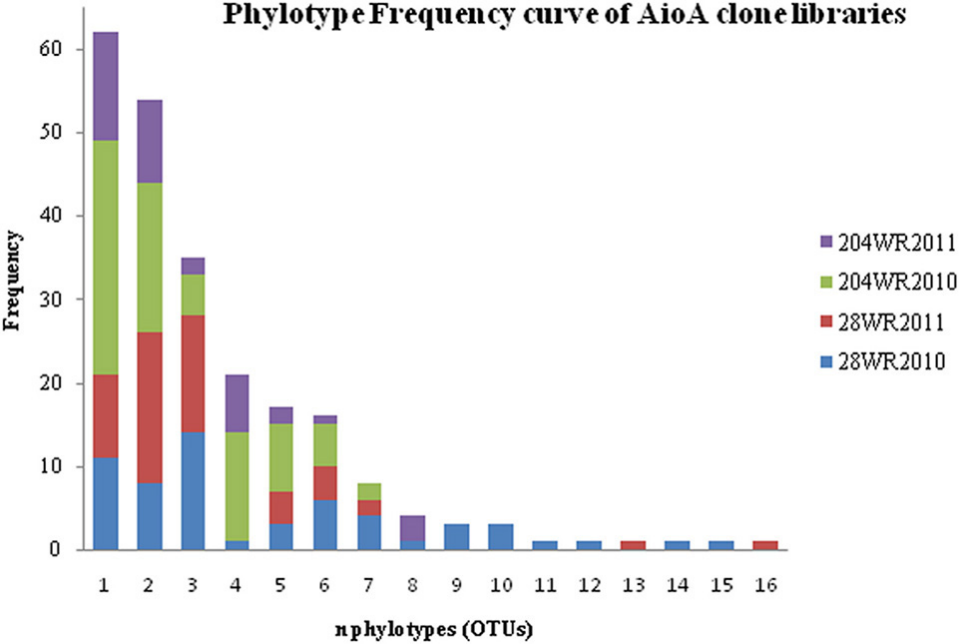
**Phylotype frequency curve for 229 *aioA* sequences distributed among 16 phylotypes**.

Based on OTU calculations, all 229 *aioA* clones were grouped into 16 operational taxonomic units (OTUs), with a cut-off of 2% at the amino acid level. The 28WR2010 *aioA* clone library had the highest number of OTUs, followed by three other *aioA* clone libraries namely, 28WR2011, 204WR2010 and 204WR2011.The diversity indices for all four *aioA* clone libraries have been summarized in Table 3. Based on rarefaction analysis, the rarefaction curve of *aioA* clone library 28WR2010 showed under-saturation indicating more sequencing effort can possibly detect rare sequences, but the rarefaction curves of other three *aioA* clone libraries 28WR2011, 204WR2010 and 204WR2011 reached an asymptote, implying that the sequencing effort was optimal (Figure 4). The H′ index for all *aioA* clone libraries combined was 2.03. The H′ index of 2010 *aioA* clone libraries 28WR2010 (H′ = 2.16) and 204WR2010 (H′ = 1.71) were higher compared to 2011 *aioA* clone libraries 28WR2011 (H′ = 1.68) and 204WR2011 (H′ = 1.60). The overall richness for all *aioA* clone libraries combined was found to be 31. The *aioA* clone library 28WR2010 showed a higher Chao 1 value (23) compared to the other three *aioA* clone libraries i.e., 28WR2011 (Chao 1 value 8.5), 204WR2010 (Chao 1 value 7) and 204WR2011 (Chao 1 value 7). The phylotype frequency curve of all *aioA* clone libraries combined indicated that 65% of these sequences were represented by 4 common OTUs under the order *Burkholderiales* of *Betaproteobacteria.* These were represented by *Acidovorax* like *aioA* clone sequences (1 OTU), *Hydrogenophaga* like *aioA* sequences (2 OTUs) and *Albidiferax* like *aioA* clones (1 OTU). About 28% of the sequences were represented by 3 common OTUs of *Alphaproteobacteria* belonging to *Rhodobacterales* (2 OTUs) and *Rhizobiales* (1 OTU). Only 5% of the sequences were represented as singletons (8 OTUs) of which 6 OTUs belong to the *aioA* clone library 28WR2010 (Figure 5).

**Table 3:** **Comparison of diversity indexes among *aioA* gene and 16S rRNA related sequences between the two wells 28 and 204 for the two years (2010 and 2011)**.

	**Name of the clone library**									
	**Total *aioA* library**	**BDP28 WR2010**	**BDP28 WR2011**	**BDP204 WR2010**	**BDP204 WR2011**	**Total 16S rRNA library**	**BDP28W S2010**	**BDP28W S2011**	**BDP204 S2010**	**BDP204 S2011**
Number of Sequences	229	58	54	61	56	169	46	38	43	42
Number of OTU^*^	16	13	8	7	7	18	10	6	11	9
Shannon index (H′)	2.023	2.167	1.683	1.717	1.601	2.229	1.711	1.383	2.149	1.914
Chao1 estimation	31	23	8.5	7	7	21	12	7	14	9

* aioA OTUs were defined at 2% amino acid divergence; 16S rRNA OTUs were defined at 3% nucleotide divergence.

**Figure 6:**
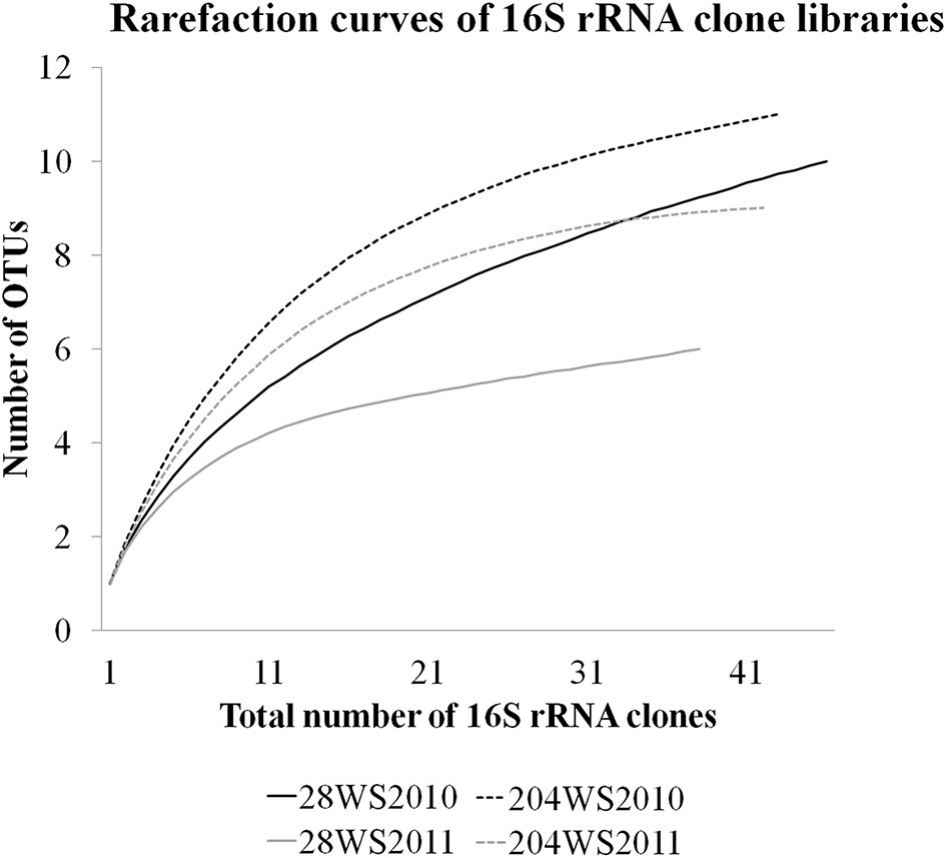
**Rarefaction curves from DOTUR analysis using furthest neighbor assignment algorithm with the 16S rRNA gene sequences retrevied from wells 28 and 204 for the two years (2010 and 2011) at 0.03 evolutionary distance**.

**Figure 7:**
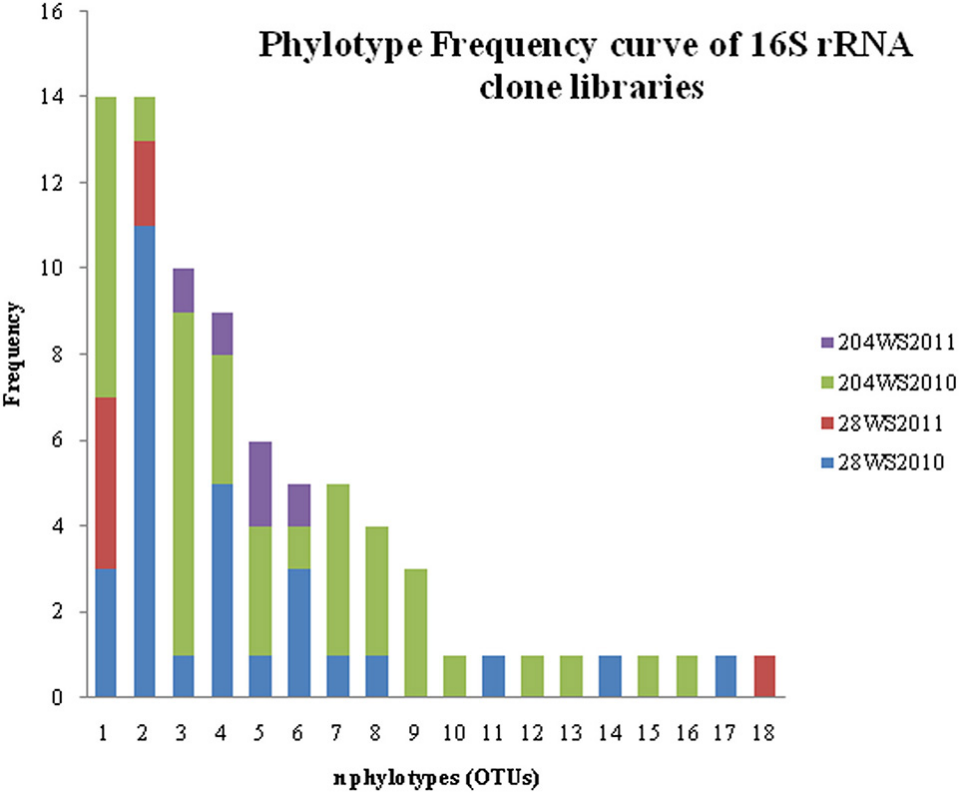
**Phylotype frequency curve for 169 16S rRNA sequences distributed among 18 phylotypes**.

In DOTUR analysis of 169 16S rRNA clones, all the sequences grouped into 18 OTUs, with a cut-off of 3% at the nucleotide level. In year 2010 samples, 16S rRNA clone libraries 204WS2010 and 28WS2010 had the highest number of OTUs, followed by 204WS2011 (Table 3). Lowest number of OTUs was recorded in 28WS2011. The diversity indices of all four 16S rRNA clone libraries combined have been summarized in Table 3. The comparative OTU richness within the four 16S rRNA clone libraries was studied by rarefaction analysis. The rarefaction analysis of 2010 libraries (28WS2010 and 204WS2010) showed under saturation suggesting that further sequencing effort was necessary. In contrast, the rarefaction curves of 16S rRNA clone libraries from 2011 sampling event 28WS2011 and 204WS2011 showed an asymptote implying that the sequencing effort was optimal (Figure 6). The H′ index for all 16S rRNA libraries combined was found to be 2.22. The index was highest for 204WS2010 library followed by 204WS2011, 28WS2010 and 28WS2011, respectively (Table 3). An overall OTU richness for all the four 16S rRNA libraries combined and measured with the Chao 1 estimator was 21. The libraries generated from 2010 sampling session 28WS2010 and 204WS2010 had higher Chao 1 values compared to 2011 clone libraries (28WS2011 and 204WS2011). In the phylotype frequency curve based on total number of 16S rRNA clones generated in this study, 61.5% of the sequences were represented by 5 common OTUs of *Betaproteobacteria* belonging to *Rhodocyclale* (3 OTUs) and *Burkholderiales* (2 OTUs) (≥1 sequence from each clone library per OTUs), whereas 10% of sequences were represented as singletons (8 OTUs) out of which 7 OTUs were represented in 204WS2010 clone library (Figure 7).

### Principal component analysis

The PCA analysis related *aioA* gene OTUs to have a cumulative % variance for the first two PCs as 86.6% (PC1- 55.9% and PC2- 30.7%). As evident from the loadings of PC1 (Table S4 in supporting information), Na, Mg, Fe, K and As had greater influence than other elements on *aioA* gene OTU distribution, whereas Na, Fe and As have negative regulatory roles. Notably As was closer to the cumulative point than other elements. The plot (Figure S3 in supporting information) clearly showed that the OTUs generated from the two stations at two different time points were independent of each other. The PC2 was mainly described by the high loadings of ionic conductivity and TDS.

The cumulative % variance in PCA analysis related to 16S rRNA OTUs for the first two PCs was 88.8% (PC1-57.1% and PC2- 31.6%). The influence of Na, Mg, Fe, K and As on 16S rRNA OTU distribution was explained by the loadings of PC1. However, Mg and Mn showed a negative influence on 16S rRNA OTU distribution. The PC2 was explained by TDS, ionic conductivity and the negative effect of Si concentration.

## Discussion

### Distribution of trace metals in groundwater

The biogeochemical cycling of As is closely related to the biogeochemical cycling of other elements such as Fe, Mn, N, S and Cl (Edwards and Santini, 2013). Chemically and microbially mediated oxidation-reduction processes influence the dissolved As level in different ecological niches. Many hypotheses have been proposed (Bhattacharya et al., 1997, 2001; Nickson et al., 2000; McArthur et al., 2004) to explain As contamination in BDP aquifers, of which microbial reductive dissolution of As bearing Fe(III) elements is most widely accepted. However, our knowledge is limited about the major bacterial groups carrying out different oxidation/reduction reactions, which play a role in distribution of As in BDP aquifers.

To study the effect of variable precipitation following the end of monsoon and/or recharge into these wells, we compared the change in major elemental concentrations between the two sampling events. We observed increase in concentrations of As, Fe, S, Si and Na (Table 2) in both wells in 2011, when precipitation is less, suggesting pre-concentration of inorganic elements due to less rainwater dissolution of elements. However, elements like Mg, Mn, Mo and K do not increase in concentration, and may imply that besides precipitation there are other processes, which have an effect on elemental distribution (i.e., elements such as As, Fe, S, Si and Na). Chemical and biological reductive dissolution of As bearing iron oxy-hydroxides and sulfidic elements in these shallow aquifers can be one of the likely reasons for increase of As, Fe and S concentrations (Acharyya et al., 2000; McArthur et al., 2004; Pedersen et al., 2004; Erbs et al., 2010). We observe enrichment of P in 2010 samples, which may be due to addition of phosphate fertilizers during the month of August for paddy cultivation (according to local farmers). It has also been reported that As co-exists with Si and P in iron-hydroxide suspension in groundwater (Zeng, 2004). Thus, depletion in P level can be due to competitive adsorption of P to iron-hydroxides, which contribute toward increase in As levels in groundwater (Acharyya et al., 2000; Bang and Meng, 2004).

The increase in ionic conductivity observed during 2011 suggests enhanced degradation of organic compounds (Routh et al., 2001; Atekwana et al., 2004). The BDP region has always been strongly influenced by monsoon, recharge of surface water and *in situ* microbial processes, which contribute to the increase of DOC (Ghosh et al., 2014) and ionic conductivity in groundwater. While previous studies had suggested bacterial decomposition of organic matter in sediments and groundwater as the primary reason for release of Fe and As in these aquifers (Islam et al., 2004; McArthur et al., 2004), but due to lack of evidence this idea it remains in-conclusive. Ongoing investigations at this site indicate diverse microbial communities and high inputs of DOC derived from microbial decomposition of organic matter in these wells (Ghosh et al., 2014). In this study, we have reported presence of bacterial communities and their metabolic processes affecting As and other dissolved metal fluxes in BDP groundwater (see below).

### Validation of the designed primer set

The amino acid sequence of arsenite oxidase large sub-unit *aioA* protein had a CX2CX3CX_70_S (CHFCIVGCGYH) motif for [3Fe-4S] binding and another consensus motif YEKGIIWGNDN. Earlier studies (Quemeneur et al., 2008) had selected these two consensus regions to design degenerate primers. In this study, we targeted a smaller nucleotide stretch (18 bp) of the same [3Fe-4S] binding motif to design the forward primer *aioA*F. To design the reverse primer, we targeted the consensus motif IIWGNDN. In this motif, the first isoleucine can be replaced by leucine or phenyl-alanine with a change in the first nucleotide of the codon CUC/UUC/AUC (5 to 3′), respectively as reported in published *aioA* sequences. To avoid degeneracy while designing the reverse primer *aioA*R, we removed the 3′ base at wobble position of the anti-codon, which gives a shorter nucleotide stretch of 18 bases. The newly designed primer set can therefore target a wider range of bacterial phyla including environmental samples, which is a significant methodological improvement in this study. As discussed in the result section, the new primers were bacterial *aioA* specific and successfully amplified *aioA* amplicons from all the arsenite oxidizing bacterial strains, many of which have been reported previously in published literature. Moreover, the primers did not amplify any of the non-arsenic oxidizing bacteria confirming the specificity of primers. Additionally, except *aioA* gene related sequences, there is complete absence of non-specific amplicons from the environmental clone libraries, which indicate that the newly designed primes were highly specific for bacterial *aioA* related sequences. Although the extant *aioA* primers were more degenerative, they can be biased at times (Quemeneur et al., 2008) targeting a narrow range of arsenite oxidizing bacteria. However when these primers were tested on BDP environmental DNA no amplicons were generated.

### Comparison of bacterial community structure

In this study, we primarily focused on the assemblage of *in situ* bacterial flora and As(III) oxidizing bacterial assemblages in shallow gray sand As-rich aquifers. The availability of nutrients and effect of increasing As concentration due to higher precipitation in 2010 showed an effect on bacterial community structure and diversity. Although previous studies identified a wide phyletic distribution of bacterial *aioA* sequences across different geographical realms, in this study, we detected *aioA* sequences showing close taxonomic affiliations only with *Proteobacteria*. Likewise, bacterial16S rRNA diversity in this study shows a similar pattern with the dominance of *Proteobacteria*-like sequences based on the number of clones sequenced. The mesophilic temperature in groundwater (Table 1) could be one of the reasons for this trend. Moreover, earlier reports indicated that 98% of bacterial *aioA*-like sequences generated from mesophilic sediments belong to *Proteobacteria* (Inskeep et al., 2007). An overwhelming predominance of *Betaproteobacteria*-like *aioA* sequences occured in both wells, which was in agreement with previous studies based on the phylogeny of arsenite oxidase large sub-unit (Quemeneur et al., 2008; Salmeron et al., 2011).

The bacterial *aioA* clone libraries from both wells primarily consisted of *aioA*-like sequences under the two classes *Alphaproteobacteria* and *Betaproteobacteria*, with dominant bacterial genera namely, *Hydrogenophaga*, *Acidovorax, Albidiferax, Bosea*, and *Polymorphum* in the aquifers. Between the two sampling events, variation in As(III) oxidizing bacterial community assemblages was clearly evident in well 28. In comparison to the *aioA* clone assemblages in library 28WR2010, there was a decrease in the number of *aioA* clones showing identity to *aioA* amino acid sequences belonging to the bacterial genera *Acidovorax*, *Albidiferax* and *Bosea*, and an increase in *Hydrogenophaga*-like *aioA* clones from library 28WR2011 (Figure 2). However, in well 204 observable variations among the two *aioA* clones libraries were much less. We observed a decrease in the number of *aioA* clones showing significant identity with bacterial *aioA* sequences belonging to the genera *Albidiferax, Bosea* and *Polymorphum* from library 204WR2011 (Figure 2).

Previous investigations indicated presence of petroleum derived hydrocarbons within the clay lenses in gray sand aquifers as an important source of carbon available for microbial uptake (Rowland et al., 2006). Consistent with this, we detected presence of such hydrocarbons in the lipid fraction extracted from groundwater DOC and aquifer sediments (Ghosh et al., 2014). Interestingly, we observed that among the As(III) oxidizing bacterial assemblages, 67 *aioA* clones (29.2%) cluster with *aioA* sequence of crude oil degrading bacterium *Polymorphum gilvum* SL003B-26A1 (Nie et al., 2012). Moreover, we also detected presence of *Acinetobacter* sp. like 16S rRNA clones (26 clones; 15.3%; Figure S2b), which researchers have reported as a crude oil degrading genus (Lal and Khanna, 1996). The presence of these clones was noteworthy because they indicates bacterial degradation of complex organic substrates in the BDP sediments, which consist of *in situ* organic matter (e.g. lignite), petroleum derived compounds, and/or allochthonous organic matter transported by rainwater from distant sources playing a crucial role in As cycling (McArthur et al., 2004; Rowland et al., 2006).

Based on sequencing, the 16S rRNA libraries were dominated by *Proteobacteria*-like sequences, which included *Alphaproteobacteria*, *Betaproteobacteria* and *Gammaproteobacteria*. Other than the phylum *Proteobacteria*, we also encountered *Nitrospirae*. Amongst the two 16S rRNA clone libraries generated from well 28, in comparison to the library 28WS2010, there was an observed decrease in the number of *Burkholderiales* and *Pseudomonadales*-like 16S rRNA clones in library 28WS2011. The assemblage of *Rhodocyclales* increased in this well between the two sampling events. We observed heterogeneity in the 16S rRNA clones in well 204, where the number of 16S rRNA clones under the bacterial order *Burkholderiales* increased in library 204WS2011 in comparison to 204WS2010. However, the assemblage represented by *Pseudomonadales* and *Rhodocyclales*-like sequences decreased between the sampling events. Heterogeneity in the clone libraries was also observed among the 16S rRNA phylogeny, where *Alphaproteobacteria*-like16S rRNA sequence detected showed close similarity (99%) with 16S rRNA of the nitrite and selenite reducing bacterium *Rhizobium selenitireducens* strain B1 (detected only in 204WS2010). In addition, we detected phylum *Nitrospirae* in well 204, where 4 16S rRNA clone sequences (from the library 204WS2011) showed 100% identity with 16SrRNA of iron-oxidizing bacterium *Leptospirillum* sp. E4-L9.

Many As(III)oxidizing bacterial genera under order *Burkholderiales*, which were represented in *aioA* assemblages, also occur in the 16S rRNA clone libraries. These included the genera *Acidovorax*, *Hydrogenophaga* and *Variovorax*. Some sequences showed significant identities with published As(III) oxidizing bacterial genera only in 16S rRNA libraries, but not in the *aioA* clone libraries like the genera *Acinetobacter* and *Rhizobium*. Thus, ca. 21% of the 16S rRNA clones in this study clustering with Burkholderiales were similar to As(III) oxidizing bacterial groups. In addition to arsenite oxidizing bacterial groups, we also detected arsenate reducing bacteria (17%). Among the 16S rRNA sequences, we detected sequences having identity (99%) with known arsenate reducing bacterial genera *Curvibacter* sp., a putative symbiont of *Hydra magnipapilata* (Chapman et al., 2010) and *Acinetobacter* (Anderson and Cook, 2004; Routh et al., 2005). Thus, more than one third of the bacterial 16S rRNA clones sequenced and identified in this study were found to be similar to bacterial strains that have been associated with As biogeochemical cycling.

Fe(II) oxidized by chemical or biological processes strongly adsorbed As and removed them from water (Katsoyiannis and Zouboulis, 2006). Bacteria participating in oxidation/reduction reactions in Fe cycling were also co-linked to As biogeochemical cycling (Meyer-Dombard et al., 2012). Presence of high nitrate levels in these aquifers indicated possible contribution of agricultural runoff (data not shown). In this context, we reported dominance of the bacterial genus *Acidovorax* in both *aioA* and 16S rRNA assemblages and *Dechloromonas* in 16S rRNA assemblages. *Acidovorax* and *Dechloromonas* have been described as potent nitrate dependent-Fe(II) oxidizers (Chakraborty and Picardal, 2013). Moreover, we detected clones in the 16S rRNA assemblages showing 100% identity with 16S rRNA sequence of *Leptospirillum*, a Fe(II) oxidizer. Along with these Fe(II) oxidizing bacteria, we also detect *aioA* clones clustering with *aioA* sequence of Fe(III) reducing bacterium *Albidiferax ferrireducens* (earlier named *Rhodoferax ferrireducens*; Finneran et al., 2003) in the *aioA* phylogenetic tree (Figure S1 in supporting information). Moreover, we also detected 16S rRNA clones clustering with 16S rRNA of Fe(III) reducing bacterium culture clone HN104 (Acc. No. FJ269070; Figure S2a in supporting information).The Fe(III) reducing As(III) oxidizing bacteria have been suggested to play an important role in the biogeochemical cycling of Fe and As through reductive dissolution of As bearing Fe(III) oxides and subsequent mobilization of As (Nickson et al., 1998, 2000; McArthur et al., 2001, 2004) in these aquifers, but microbiological evidence was missing. Thus, detection of these Fe(III) reducing bacteria like 16S rRNA sequences provides evidence for reductive dissolution processes involving arseniferous iron-oxyhydroxides, and solubilizing Fe and As in BDP aquifers.

Oxidation of As cannot solely depend on the presence of dissolved oxygen (DO) in these anoxic groundwater because oxygen is rapidly consumed by sulfidic elements, other reducing compounds and oxidation of organic matter. Freshly recharged groundwater contains more DO, however it is quickly utilized and sets in anoxia during late monsoon. Thus, alternate redox reactions were likely in these aquifers. In fact, several studies have indicated that bacteria can utilize alternate oxidants to gain energy e.g., As(III) oxidation (Oremland and Stolz, 2003) and Fe(II) oxidation (Straub et al., 2001). Likewise, bacterial chlorate (ClO^−^_3_) reduction to generate energy in enrichment cultures from sludge samples and pure cultures of *Dechloromonas* sp. using ClO^−^_3_ as an electron acceptor supports bacterial oxidation of As(III) (Sun et al., 2010). In this study, we reported 16S rRNA clones from the library 28WS2010, 28WS2011, and 204WS2010, which had 100% identity with *Dechloromonas* sp. enrichment culture clone ECC3-2 (Acc. No. GU056291). The microbiological evidence suggested the possibility of chlorate dependent As(III) oxidation in BDP aquifers (Sun et al., 2010). These alternate oxidants perhaps play an ecologically significant role to support anoxic bacterial processes, and control the distribution of As in BDP aquifers.

Notably, 3 identical Burkholderial 16S rRNA clones BDP204WS2, BDP204WS3, and BDP204WS10, showed 96% identity with the 16S rRNA sequence of *Acidovorax* sp. KKS102 (Acc. No. CP003872) cluster separately (Figure 3). Similarly, other 3 Burkholderial 16S rRNA clones BDP204WS23, BDP204WS24, and BDP204WS227 showed 97% identity with the 16S rRNA sequence of an uncultured bacterium strain 179up (Acc. No.AY212630) that clustered separately (Figure S2a in supporting information). The data indicated presence of novel As(III) oxidizing bacterial genera in BDP aquifers, which have not been reported in published studies to date.

### Statistical representation of phylogenetic diversity

Of the 229 *aioA* clone sequenced, *Betaproteobacteria* were represented by 4 common OTUs and *Alphaproteobacteria* were represented by 3 common OTUs. The clone library 28WR2010 had higher number of OTUs (13) and higher H′ index compared to other *aioA* clone libraries. During the next sampling event, the number of OTUs reduced to 8 along with a fall in H′ index (Table 3), whereas in phylotypes, there were 14 common OTUs detected in the clone library 28WR2010, 6 OTUs in 28WR2011. We also saw under saturation in rarefaction of the 28WR2010 library. However, rarefaction in 28WR2011 library was saturated, indicating loss of bacterial diversity under the influence of increasing As concentration (Figure 5). Comparatively less statistically significant loss in As(III) oxidizing bacterial diversity was observed in well 204, where the number of OTUs and the number of phylotypes among the two libraries 28WR2010 and 28WR2011 remained the same (7), but H′ index reduced by 0.1 units. The changes in terms of species richness, between the two sampling events could be further investigated using the non-parametric species richness estimator Chao 1. Thus, increasing concentrations of different elements including As and Fe showed a possible negative effect on the species richness in well 28. The high Chao 1 value (23) in library 28WR2010 reduced to 8.5. We did not see such effect on As(III) oxidizing bacterial richness in clone libraries generated from well 204 (Table 3).

Of the 169 16S rRNA clones sequenced, *Betaproteobacteria* were represented by 5 common OTUs. The libraries generated from 2010 sampling event (i.e., 28WS2010 and 204WS2010) showed greater bacterial diversity in terms of the number of OTUs and H′ indices (Table 3). However, 28WS2010 had greater number of phylotypes (10), which reduced in library 28WS2011 to 6. A similar effect was observed in well 204, where in library 204WS2010 there were 14 different phylotypes, which reduced to 10 in library 204WS2011. The under saturation in rarefaction of these two libraries indicated more efforts in terms of sequencing might be necessary (Figure 7). The bacterial diversity measured with H′ index and bacterial richness measured by Chao 1 reduced during the next sampling event in 2011 in both the wells, with saturation in their rarefaction curves. Thus, an overall effect of monsoon through precipitation input and recharge on the diversity and richness of the whole bacterial community studied based on 16S rRNA sequences seemed to be evident from this study.

Although the PCA analysis was limited by the number of samples analyzed as part of this study, it showed that while the OTUs were independent, the loadings depicted that there was an effect of metals, especially As on influencing bacterial diversity (16S rRNA OTUs). However, less effect was seen on *aioA* OTUs, which may imply the adaptability of As(III) oxidizing bacteria in response to stress. In future, thorough sampling efforts (in terms of number of samples collected) followed by analysis of microbial communities in BDP aquifers with special reference to elemental concentrations before and after monsoon can be undertaken, and subsequently robust statistical analyses could be employed to gain an understanding of the role of environmental variables in shaping the community structure of arsenite oxidizing bacterial phyla.

Although further sequencing effort is warranted along with increased sampling intensity, nevertheless trends emerging from this study showed that bacterial communities present in both wells seemed to be affected by changes in physical and chemical parameters after monsoon, which was most likely induced by variation in precipitation. Based on *aioA* and 16S rRNA clone library and sequencing approach, it seemed that these aquifers have an unusually low bacterial diversity, where *Proteobacteria*-like sequences dominated in both the wells and their community composition was influenced by varying concentration of As as well as other elements during both sampling events. However, to get a better understanding of absolute diversity trends for bacterial communities in these aquifers, multiple sampling events all year round is warranted. The temporal trend of *aioA* and 16S rRNA based bacterial community structure, varied mostly due to their location and usage. Well 28 being located in a household, was utilized less. However, well 204 being located near an agricultural field it is used for irrigation. Although both wells are located on GSA, yet they are heterogeneous in terms of sedimentary organic matter content (Ghosh et al., 2014), which also explained their heterogeneity in terms of bacterial community structure including arsenite oxidizing bacterial phylotypes. Our study showed that many of the *aioA* and 16S rRNA bacterial sequences identified from this area have been previously reported to play major role in elemental cycling (including As). The presence of crude-oil degrading bacterial *aioA* and 16S rRNA sequences in our libraries highlight their importance in degradation of hydrocarbons present in DOC and sediments aquifer. More interestingly, we encountered several novel lineages of bacteria-like 16S rRNA sequences in both aquifers, which harbor unidentified bacterial genus that may play key role in biogeochemical cycling of As, Fe, and other elements. Overall the *aioA* and 16S rRNA sequences generated from the BDP aquifers provide us with a firsthand insight on functional diversity of bacterial communities, and their inter-annual variability in connection with dissolved elemental concentrations.

Several studies as discussed before in the past had focused on geochemical aspects of As contamination in Bengal aquifers. In these studies/reports scientists had repeatedly mentioned about *in situ* bacterial population which play a major role in As biogeochemical cycling mainly in terms of indirect geochemical evidence. To the best of our knowledge, bacterial community structure based on arsenic metabolizing genes has mostly been carried out in extreme environments like hydrothermal vents, acid mines (Oremland and Stolz, 2005), but rarely in aquifers. These extreme environments are vastly different in their physical, chemical and biological characteristics than groundwater aquifers. Thus, a critical knowledge gap exists in our understanding the microbial community structure and the arsenic metabolizers, which exist in these aquifers and how they drive As cycling. In this context as pointed out earlier in our manuscript, this study in the Bengal aquifers is one of the first of its kind, where arsenite oxidizing bacterial community structure has been investigated based on the functional gene sequencing approach along with targeting of 16S rRNA marker. We have provided evidence of these microbial communities which play a role in As cycling in BDP aquifers. Additionally, the sequences generated from this study provides additional direction in comparing the global distribution pattern of arsenite oxidizing bacterial communities in other As impacted regions (e.g., Bangladesh, Vietnam, Argentina, USA, Bolivia) and understanding the biogeochemical aspects which impact the survival of these microbes.

Future work will focus on the study of diversity of cultivable As(III) oxidizing bacteria based on culture approaches so that the isolates can be utilized for *in situ* bioremediation of As in contaminated aquifers as part of an ongoing pilot project and providing safe drinking water to the local community living in BDP region. The detection of arsenite oxidizing bacterial *aioA* sequences in BDP aquifers are indicative of their presence in this type of environment, however to understand the functional significance of these bacteria in As cycling future studies could be focused on quantitative PCR approaches where *aioA* transcript numbers can be quantified and subsequently linked to changing elemental concentrations in aquifers water. Moreover, the role of available organic carbon sources in sustaining these bacterial groups in BDP aquifers can be also investigated as part of future investigations.

### Conflict of interest statement

The authors declare that the research was conducted in the absence of any commercial or financial relationships that could be construed as a potential conflict of interest.

## Footnotes

^1^http://www.wbphed.gov.in/main/Static_pages/ArsenicReport/blockwise%20arsenic_a4.pdf

^2^http://www.imd.gov.in/section/hydro/distrainfall/webrain/wb/nadia.txt
