# Supplementary material for: Diversity of arsenite oxidizing bacterial communities in arsenic-rich deltaic aquifers in West Bengal, India
Source: Front Microbiol. 2014 Nov 21;5:602. doi: 10.3389/fmicb.2014.00602 (PMC4240177; doi:10.3389/fmicb.2014.00602)
Supplement: Supplementary file 1 [file DataSheet1.DOCX]

**Table S1**

Percentage of Arsenic affected (As content in mg/l) tube wells of Block Karimpur II (**Report based on Water Quality Survey during 2002-2006 under the joint plan of action of** PHED, Govt. of W.B. and **UNICEF**)

| **Arsenic content in mg/l** | | | | | | Total number of tube wells |
| --- | --- | --- | --- | --- | --- | --- |
| <= 0.01 | | 0.01<= 0.05 | | >0.05 | |  |
| Percentage | Count | Percentage | Count | Percentage | Count | 1,097 |
| 26.57% | 447 | 27.64% | 469 | 45.96% | 780 |  |

**Table S2**

| AioA Clone Library | Highest identity at the amino acid level | | | Lowest Identity at the amino acid level | | |
| --- | --- | --- | --- | --- | --- | --- |
|  | Maximum identity in pblast | Description of *aioA* sequence showing closest match | Clone name | Maximum identity in pblast | Description of *aioA* sequence showing closest match | Clone name |
| 28WR2010 | 99% | Uncultured bacterium *aioA* clone (Acc. No.BAN63590) | BDP28WR29, BDP28WR30,  BDP28WR32, BDP28WR47, BDP28WR48, BDP28WR49, BDP28WR50, BDP28WR52, BDP28WR53, BDP28WR55 | 90% | Uncultured bacterium *aioA* clone (Acc. No. BAN63553) | BDP28WR2, BDP28WR18,  BDP28WR21, BDP28WR26 |
| 28WR2011 | 99% | Uncultured bacterium *aioA* clone (Acc. No.BAN63590) | BDP28WR66, BDP28WR68, BDP28WR79, BDP28WR80, BDP28WR89, BDP28WR90 , BDP28WR91, BDP28WR94 , BDP28WR96, BDP28WR97, BDP28WR98, BDP28WR100 , BDP28WR103, BDP28WR112 | 83% | Uncultured bacterium *aioA* clone (Acc. No. ABY19344) | BDP28WR77 |
| 204WR2010 | 99% | Uncultured bacterium *aioA* clone (Acc. No.BAN63590) | BDP204WR16 , BDP204WR42, BDP204WR56, BDP204WR57 | 90% | Uncultured bacterium *aioA* clone (Acc. No. BAN63553) | BDP204WR20 BDP204WR55 |
| 204WR2011 | 99% | Uncultured bacterium *aioA* clone (Acc. No.BAN63590) | BDP204WR74, BDP204WR75, BDP204WR115 | 90% | Uncultured bacterium *aioA* clone (Acc. No. BAN63553) | BDP204WR66 BDP204WR71  BDP204WR73 |

**Table S3**

| 16S rRNA Clone Library | Highest identity at the nucleotide level | | | Lowest identity at the nucleotide level | | |
| --- | --- | --- | --- | --- | --- | --- |
|  | Maximum identity in nblast | Description of16S rRNA subject sequence showing closest match | Clone name | Maximum identity in nblast | Description of16S rRNA subject sequence showing closest match | Clone name |
| 28WS2010 | 100% | Uncultured bacterium clone DR305 (Acc. No. JF429312) | BDP28WS29 | 94% | Uncultured bacterium clone KRA30+01 (Acc. No. AY081973) | BDP28WS12 |
|  |  | *Pseudomonas* sp. IM4 (Acc. No. FJ211165) | BDP28WS30 |  |  |  |
| 28WS2011 | 100% | *Pseudomonas* sp. IM4 (Acc. No. FJ211165) | BDP28WS77 | 95% | Uncultured bacterium clone D-68 (Acc. No. HQ860749) | BDP28WS52 |
| 204WS2010 | 99% | Uncultured bacterium clone eff35 (Acc. No. JN245734) | BDP204WS1 | 94% | Uncultured bacterium clone KRA30+01 (Acc. No. AY081973) | BDP204WS25,  BDP204WS26,  BDP204WS28 |
|  |  | *Acinetobacter* sp. JF32 (Acc. No. KC172007) | BDP204WS4, BDP204WS41 |  |  |  |
|  |  | Uncultured sludge bacterium A16 (Acc. No. AF234726) | BDP204WS5, BDP204WS37 |  |  |  |
|  |  | Uncultured bacterium clone MW-B26 (Acc. No. JQ088342) | BDP204WS6, BDP204WS7, BDP204WS20 |  |  |  |
|  |  | Uncultured *Acinetobacter* sp. clone TCCC 11167 (Acc. No. EU567047) | BDP204WS8, BDP204WS9, BDP204WS12, BDP204WS14, BDP204WS15, BDP204WS16, BDP204WS18 |  |  |  |
|  |  | Uncultured bacterium clone 6'-68 (Acc. No. JQ923811) | BDP204WS22, BDP204WS42 |  |  |  |
|  |  | Uncultured *Zoogloea* sp. clone W5S43 (Acc. No. GU560185) | BDP204WS29, BDP204WS33, BDP204WS34, BDP204WS35, BDP204WS40 |  |  |  |
|  |  | Uncultured bacterium clone E13 (Acc. No. EU864455) | BDP204WS38 |  |  |  |
|  |  | *Curvibacter* putative symbiont of *Hydra magnipapillata*(Acc. No. FN543107) | BDP204WS39, BDP204WS43 |  |  |  |
| 204WS2011 | 100% | *Acidovorax* sp. KKS102 (Acc. No. CP003872) | BDP204WS49. BDP204WS63 | 94% | Uncultured bacterium clone J112 (Acc. No. GQ388936) | BDP204WS44, BDP204WS48 |
|  |  | *Hydrogenophaga*sp. p3(2011) (Acc. No. HQ652595) | BDP204WS56 |  |  |  |
|  |  | *Pseudomonas* sp. IM4 (Acc. No. FJ211165) | BDP204WS58, BDP204WS60, BDP204WS62 |  |  |  |
|  |  | Uncultured bacterium clone 3BH-10FF (Acc. No. EU937983) | BDP204WS64, BDP204WS73,BDP204WS78 |  |  |  |
|  |  | *Leptospirillum* sp. E4-L9 (Acc. No. HM769767) | BDP204WS65, BDP204WS67, BDP204WS78, BDP204WS79, |  |  |  |
|  |  | Uncultured *Variovorax* sp. clone 5.17m34 (Acc. No. JN679199) | BDP204WS68 |  |  |  |

**Table S4**

|  | AioA gene PC Analysis | | | 16S rRNA PC Analysis | | |
| --- | --- | --- | --- | --- | --- | --- |
|  | Components | | | Components | | |
|  | 1 | 2 | 3 | 1 | 2 | 3 |
| Eigen Value | 1.85 | 1.02 | 0.44 | 1.85 | 1.03 | 0.365 |
| Variation | 55.9 | 30.7 | 13.4 | 57.1 | 31.6 | 11.2 |
| Cumulative % variance | 55.9 | 86.6 | 100.0 | 57.1 | 88.8 | 100.0 |
| pH | -0.034 | -0.011 | 0.021 | 0.035 | -0.002 | -0.021 |
| Ionic Conductivity | -0.33 | 0.463 | -0.357 | 0.261 | 0.497 | 0.515 |
| TDS | -0.33 | 0.463 | -0.357 | 0.173 | 0.369 | 0.412 |
| Temperature | -0.226 | 0.35 | -0.294 | -0.077 | 0.035 | -0.037 |
| As | **0.072** | 0.05 | 0,028 | **0.04** | 0.042 | -0.037 |
| Fe | **-0.3** | 0.061 | 0.474 | **0.298** | 0.157 | -0.468 |
| Mg | **0.35** | 0.298 | 0.014 | **-0.38** | 0.218 | -0.014 |
| Mn | **0.053** | 0.016 | 0.147 | **-0.51** | 0.015 | -0.166 |
| Mo | 0.0 | -0.002 | -0.008 | 0.0 | -0.002 | 0.009 |
| P | -0.12 | 0.026 | 0.083 | -0.12 | 0.006 | -0.103 |
| S | -0.215 | -0.061 | 0.056 | 0.221 | -0.01 | -0.042 |
| Si | -0.191 | -0.715 | -0.477 | 0.264 | -0.692 | 0.428 |
| K | 0.244 | -0.009 | 0.044 | -0.238 | -0.058 | -0.083 |
| Na | **-0.64** | -0.064 | 0.378 | **0.646** | 0.101 | -0.336 |
| Respective OTUs* | **0.225** | -0.217 | 0.392 | **-0.223** | 0.221 | -0.003 |

^*^AioA OTUs for AioA gene PCA analysis; 16S rRNA OTUs for 16S rRNA PCA analysis
