# Supplementary figures and images for: Diversity of arsenite oxidizing bacterial communities in arsenic-rich deltaic aquifers in West Bengal, India
Source: Front Microbiol. 2014 Nov 21;5:602. doi: 10.3389/fmicb.2014.00602 (PMC4240177; doi:10.3389/fmicb.2014.00602)

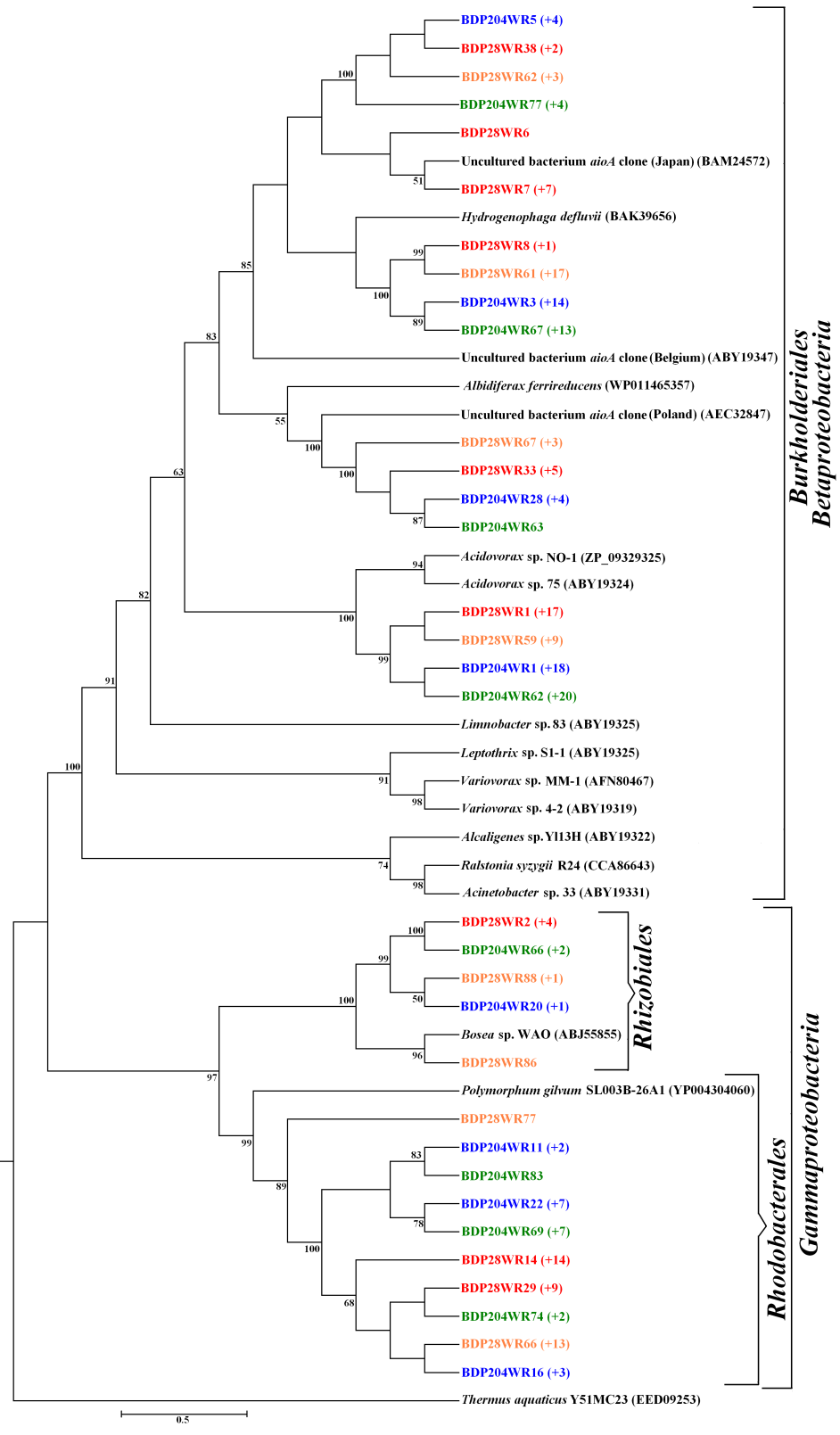


**Fig. S1**


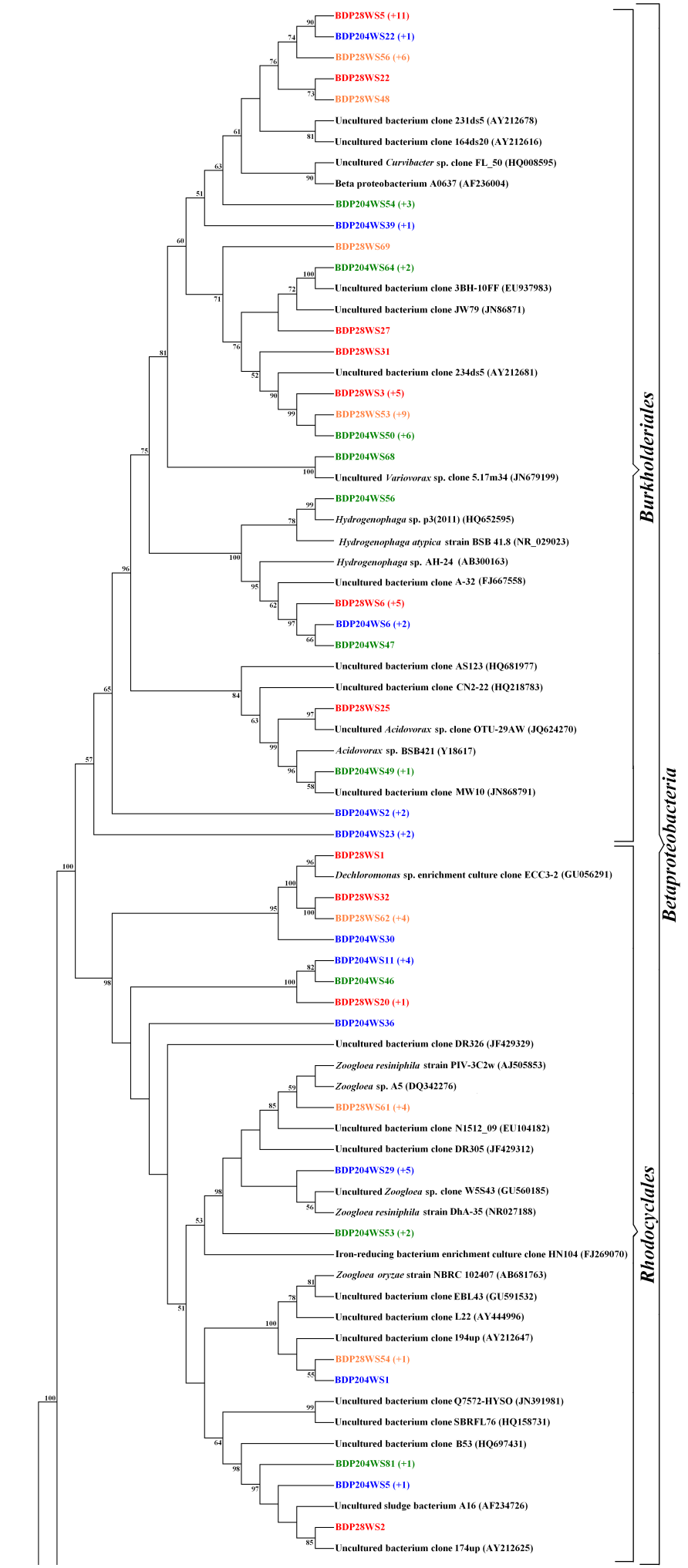


**Fig. S2(a)**


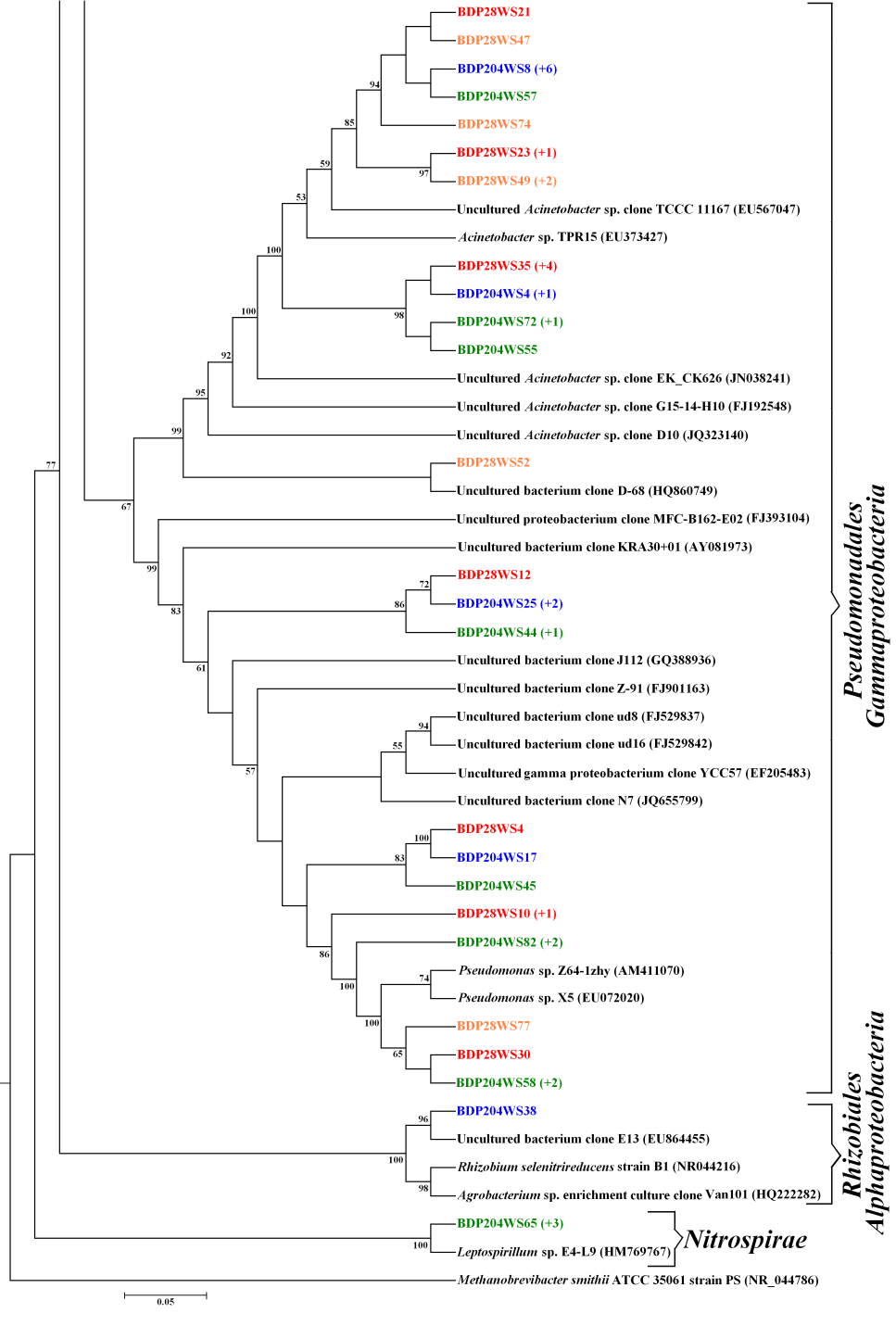


**Fig.S2(b)**

**
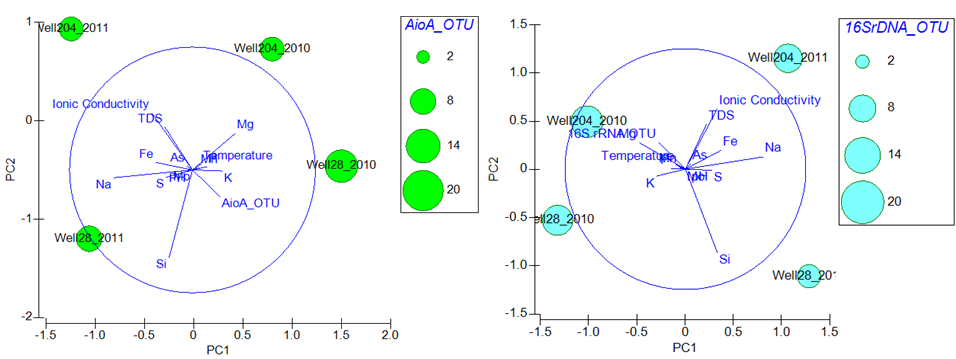
**

**Fig. S3**

Supplement: Supplementary file 2 [file DataSheet2.DOCX]
